# Factors associated with fatal coronavirus disease 2019 infections among cancer patients in the US FDA Adverse Event Reporting System database

**DOI:** 10.2217/fon-2021-0816

**Published:** 2021-11-02

**Authors:** Omar Abdel-Rahman

**Affiliations:** 1 Department of Oncology, University of Alberta, Cross Cancer Institute, Edmonton, Alberta T6G 1Z2, Canada

**Keywords:** cancer, COVID-19, mortality, outcomes

## Abstract

**Aim:**

To explore factors affecting coronavirus disease 2019 (COVID-19) mortality among cancer patients based on a pharmacovigilance database.

**Methods:**

US FDA Adverse Event Reporting System (FAERS) quarterly data extract files were reviewed for quarters two, three and four of 2020 (i.e., April to December). Patients with an indication related to malignancy and a reported COVID-related reaction were selected. Multivariate logistic regression analysis for factors associated with a fatal outcome was conducted.

**Results:**

A total of 2708 patients were included. The following factors were associated with fatal COVID-19 infection: older age (odds ratio [OR]: 1.03; 95% CI: 1.01–1.04), male sex (OR: 1.43; 95% CI: 1.07–1.91), non-US report source (OR: 2.46; 95% CI: 1.93–3.13), hematological malignancy (OR: 1.62; 95% CI: 1.28–2.07), potentially immunosuppressive treatment (OR: 1.83; 95% CI: 1.30–2.58) and diagnosis in quarter two versus quarter four (OR: 1.62; 95% CI: 1.27–2.07).

**Conclusion:**

Within FAERS reports, cancer patients who are older, males and receiving immunosuppressive treatment and those with hematological malignancies were at a higher risk of death because of COVID-19 infection.

## Lay abstract

In this study, individuals with a diagnosis of cancer who were older and males and those receiving immunosuppressive treatment seemed to be at a higher risk of a fatal outcome of coronavirus disease 2019 infection.

The coronavirus disease 2019 (COVID-19) pandemic represents one of the greatest health challenges of our time, with profound impacts on every aspect of healthcare delivery for cancer patients [1]. It was also one of the leading causes of death in the United States (and the rest of the world) in 2020 [2]. Cancer patients are known to experience higher risks of severe COVID-19 infection, particularly those on active treatment [3]. Several hypotheses have been provided to explain the more severe COVID-19 infection and higher mortality among cancer patients, including the relatively older age of cancer patients compared with noncancer patients and higher burden of comorbidity and immunosuppression experienced because of the cancer diagnosis itself and/or cancer treatment [4,5]. Within the cohort of cancer patients, it is important to identify patients at the highest risk of mortality from COVID-19 so that decisions about starting versus deferring cancer treatment in the context of expected mortality from COVID-19 can be made. The majority of patients included in prior population-based studies evaluating outcomes of COVID-19 among cancer patients were cancer survivors (i.e. not receiving active anticancer treatment). Thus, COVID-19 risk stratification among cancer patients receiving active anticancer treatment represents an unmet need. Although vaccination has provided a glimpse of hope that our healthcare systems can overcome the challenges posed by the COVID-19 pandemic, it is still unclear if the COVID-19 variants will turn this disease into a recurrent challenge similar to (or possibly more challenging than) seasonal flu [6,7].

Generation of timely, credible, real-world evidence to outline factors associated with fatal outcomes is important, as it allows policymakers to adapt to the rapidly changing nature of this pandemic. The US FDA Adverse Event Reporting System (FAERS) is a powerful tool for examining real-life treatment-associated adverse events in patients not only in the United States but also in other parts of the world, as it receives adverse event reports from different parts of the world [8]. Reporting to FAERS can be done through pharmaceutical companies, healthcare providers and the general public. Although the FDA can mandate adverse event reporting to some pharmaceutical companies as part of the approval process for a certain medicine, such reporting is done voluntarily for the majority of cases. This study utilized this tool to examine factors associated with fatal COVID-19 infection among cancer patients within the FDA pharmacovigilance database.

## Methods

### Cohort selection

FAERS quarterly data extract files were reviewed for quarters two, three and four of 2020 (i.e., April to December). The data extract files contain information about adverse events, demographics, outcomes of adverse events, types of drugs used and indication for the use of the drugs. Inclusion criteria for the current study included patients with any hematological malignancy (including myeloma, lymphoma, leukemia, plasmacytoma, myelodysplasia, myelofibrosis and myeloproliferative disorder) or solid malignancy (as identified from the indication file) and those who were reported to have COVID-19 infection (identified in FAERS according to Medical Dictionary for Regulatory Activities terms) [9]. Patients aged <18 years were excluded.

### Study variables

The following data were extracted from each report where available: age (as a continuous variable), sex, weight, country of occurrence, diagnosis of hematological versus nonhematological malignancy, type of treatment (potentially immunosuppressive vs potentially nonimmunosuppressive) and quarter of the report (two, three or four). Potentially immunosuppressive treatments include immune checkpoint inhibitors, cytotoxic chemotherapy, tyrosine kinase inhibitors, monoclonal antibodies, immunomodulatory drugs (for myeloma), proteasome inhibitors and/or CDK inhibitors. Potentially nonimmunosuppressive treatments include hormonal and supportive treatments (e.g., bone-modifying agents, pain medications). The outcome of each report, specifically whether COVID-19 infection resulted in death, was collected.

Information about concurrent use of radiation and detailed information about comorbidities are not available in the FAERS database. The exact timing of the use of any cancer treatment in relation to COVID-19 infection is also not reported in the FAERS database. Because of the small sample size of patients with hematological or nonhematological malignancies, it was not feasible to analyze the impact of individual malignancies on outcomes. Therefore, assessments were done broadly for hematological versus nonhematological malignancies. Likewise, because of the small number of individuals receiving potentially immunosuppressive or nonimmunosuppressive agents, it was not feasible to assess the impact of individual agents on outcomes. Alternatively, assessments were done broadly for patients receiving potentially immunosuppressive versus potentially nonimmunosuppressive agents. The main endpoint of the current study was COVID-19 mortality, as assessed by whether the reported outcome of the adverse event was fatal or nonfatal.

### Statistical analysis

Chi-square test and independent t-test were used to examine differences between individuals with fatal outcomes and those with nonfatal outcomes. Multivariate logistic regression analysis was conducted for factors associated with a fatal outcome. This incorporated age, weight, sex, country of occurrence, whether the malignancy was hematological, type of treatment and quarter of data release. The technique of multiple imputations was used to adjust for missing information. As a sensitivity analysis, the aforementioned model was repeated on the nonimputed dataset (excluding weight and containing only reports with complete information). All analyses were done using SPSS Statistics 23.0 (IBM Corporation, NY, USA).

## Results

### Patient characteristics

**Figure 1.**
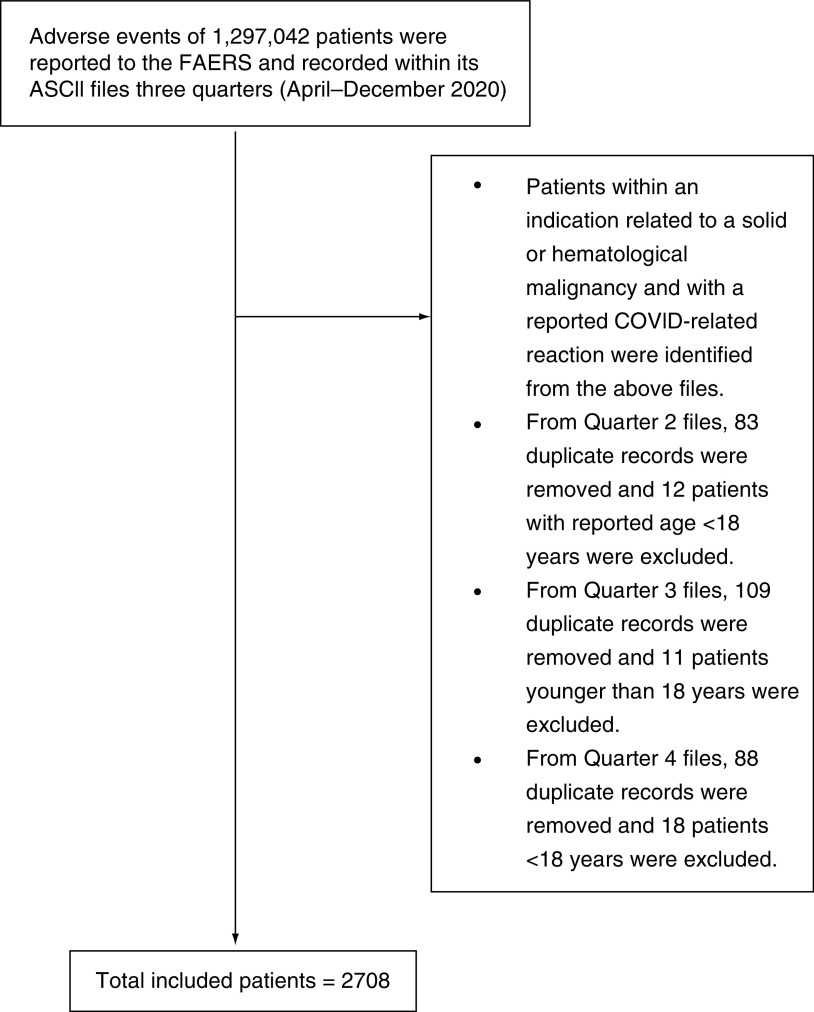
Flowchart of participant selection process. ASCII: American Standard Code For Information Interchange; COVID: Coronavirus disease; FAERS: FDA Adverse Event Reporting System.

Patients with an indication related to a solid or hematological malignancy and a reported COVID-related reaction were selected. From quarter two files, 83 duplicate records were removed and 12 patients with reported age <18 years were excluded. From quarter three files, 109 duplicate records were removed and 11 patients aged <18 years were excluded. From quarter four files, 88 duplicate records were removed and 18 patients aged <18 years were excluded. Finally, a total of 2708 patients were included (Figure 1), including 721 (26.6%) patients with fatal outcomes and 1987 (73.4%) patients with nonfatal outcomes.

Differences between individuals with or without fatal COVID-19 infection are detailed in Table 1. Patients with a fatal outcome were likely to be older (p < 0.001), males (p = 0.003) and have lower weight (p = 0.006). They were also more likely to have a source of report from outside the US (p < 0.001), hematological malignancy (p = 0.001), receipt of potentially immunosuppressive treatment (p = 0.005) and earlier quarter of data release (p = 0.001).

**Table 1.** Baseline characteristics of included cancer patients according to outcome (fatal vs nonfatal).

Parameter	Patients with fatal COVID-19 infection (n = 721)	Patients with non-fatal COVID-19 infection (n = 1987)	p-value
Age, mean (standard error)^†^	68.4 (0.58)	63.8 (0.36)	<0.001
Sex, n (%)^†^ – Male – Female	365 (58.2) 262 (41.8)	937 (51.4) 885 (48.6)	0.003
Weight, mean (standard error)^†^	74.5 (1.51)	81.5 (0.88)	0.006
Country of occurrence, n (%) – US – Non-US	292 (40.5) 429 (59.5)	1272 (64) 715 (36)	<0.001
Hematological malignancy, n (%)^¶^ – Yes – No	538 (74.6) 183 (25.4)	1352 (68) 635 (32)	0.001
Type of treatment, n (%)^‡^ – Potentially immunosuppressive – Potentially nonimmunosuppressive	624 (86.5) 97 (13.5)	1629 (82) 358 (18)	0.005
Quarter of data release, n (%)^§^ – 2 – 3 – 4	197 (27.3) 222 (30.8) 302 (41.9)	463 (23.3) 525 (26.4) 999 (50.3)	0.001

† Missing data: sex: 259; weight: 2035; age: 1123.

‡ Potentially immunosuppressive treatments include immune checkpoint inhibitors, cytotoxic chemotherapy, tyrosine kinase inhibitors, monoclonal antibodies, immunomodulatory drugs (myeloma), proteasome inhibitors and/or CDK inhibitors. Potentially nonimmunosuppressive treatments include hormonal and supportive treatments (e.g., bone-modifying agents, pain medications).

§ Q2: April to June 2020; Q3: July to September 2020; Q4: October to December 2020.

¶ Indications for treatment include leukemia, lymphoma, myeloma, myeloproliferative disorder, myelofibrosis and myelodysplasia.

COVID-19: Coronavirus disease 2019; Q: Quarter.

### Factors associated with COVID-19 mortality

The following factors were associated with a fatal COVID-19 infection: older age (odds ratio [OR]: 1.03; 95% CI: 1.01–1.04), male sex (OR: 1.43; 95% CI: 1.07–1.91), non-US source of report (OR: 2.46; 95% CI: 1.93–3.13), hematological malignancy (OR: 1.62; 95% CI: 1.28–2.07), potentially immunosuppressive treatment (OR: 1.83; 95% CI: 1.30–2.58) and diagnosis in quarter two versus quarter four (OR: 1.62; 95% CI: 1.27–2.07; Table 2).

**Table 2.** Multivariate logistic regression analysis for factors affecting fatal outcome of coronavirus disease 2019 infection^†^.

Parameter	OR (95% CI)
Age	1.03 (1.01–1.04)
Sex – Female – Male	Reference 1.43 (1.07–1.91)
Weight	0.98 (0.96–1.00)
Country of occurrence – US – Non-US	Reference 2.46 (1.93–3.13)
Hematological malignancy – No – Yes	Reference 1.62 (1.28–2.07)
Type of treatment – Potentially nonimmunosuppressive – Potentially immunosuppressive	Reference 1.83 (1.30–2.58)
Quarter of data release – 4 – 2 – 3	Reference 1.62 (1.27–2.07) 1.29 (1.04–1.61)

† Based on imputed dataset.

OR: Odds ratio.

When the model was repeated excluding weight and including only reports with complete information (1571 reports), similar results were obtained. Specifically, the following factors were associated with fatal COVID-19 infection in this cohort: older age (OR: 1.04; 95% CI: 1.02–1.05), male sex (OR: 1.30; 95% CI: 1.03–1.65), non-US source of report (OR: 1.92; 95% CI: 1.51–2.43), hematological malignancy (OR: 1.37; 95% CI: 1.07–1.76), potentially immunosuppressive treatment (OR: 1.57; 95% CI: 1.09–2.27) and diagnosis in quarter two versus quarter four (OR: 1.60; 95% CI: 1.18–2.18; Table 3).

**Table 3.** Multivariate logistic regression analysis for factors affecting fatal outcome of coronavirus disease 2019 infection among reports with complete information^†^.

Parameter	OR (95% CI)
Age	1.04 (1.02–1.05)
Sex – Female – Male	Reference 1.30 (1.03–1.65)
Country of occurrence – US – Non-US	Reference 1.92 (1.51–2.43)
Hematological malignancy – No – Yes	Reference 1.37 (1.07–1.76)
Type of treatment – Potentially nonimmunosuppressive – Potentially immunosuppressive	Reference 1.57 (1.09–2.27)
Quarter of data release – 4 – 2 – 3	Reference 1.60 (1.18–2.18) 1.23 (0.94–1.62)

† Total of 1571 reports; weight was removed from this model because it was missing from many reports.

OR: Odds ratio.

## Discussion

The results of this study are in line with previously published studies suggesting an association between COVID-19 mortality and older age, male sex and immunosuppressive therapy [10–12]. It is unclear why non-US reports have higher mortality compared with US reports. Notably, mortality seems to be decreasing with time, a possible reason for which might be related to a better understanding of COVID-19 treatment over time; however, this cannot be ascribed to vaccination, as COVID vaccination was not widely available at the time of data reporting to the FDA in 2020. Individuals with hematological malignancy have a higher probability of fatal outcomes, likely related to more immunosuppression among these individuals. The association between male sex and higher mortality is similar to previously reported data for influenza mortality among cancer patients [13]. Whether this is related to a higher comorbidity burden among men or to specific sex-based differences in response to respiratory infections is unclear.

Limitations of the study include the voluntary nature of adverse event reporting; selective reporting of more severe COVID-19 cases (more than one-quarter of patients reported in this cohort died, indicating nonreporting of mild cases); and missing data for age, weight and sex variables. To mitigate the impact of missing information on the current analysis, the technique of multiple imputation was used and the model was repeated only on reports containing complete information. Moreover, absence of information regarding comorbidity, concurrent radiation therapy and COVID-19 treatment(s) administered undoubtedly affected the veracity of the analyses within the current study. This report is also limited to individuals who were receiving active anticancer treatment at the time of COVID infection. Thus, it cannot be used to imply which factors are associated with fatal outcomes among cancer survivors who are not currently receiving active anticancer treatment. By contrast, strengths of the study include a relatively large number of reports compared with some previously published COVID-19 cancer studies and a focus on patients receiving active anticancer treatment compared with other studies that included many cancer survivors who are off anticancer treatment for many years prior to COVID-19 infection.

Prior studies have suggested that individuals with thoracic malignancies might have a higher risk of severe COVID-19 infection [14,15]. This could be related to delayed diagnosis because of similarity between thoracic cancer-related symptoms and COVID-19 symptoms and background comorbidity (e.g., cardiac or pulmonary illnesses), which are more common in these patients compared with other individuals with solid tumors. Because of the relatively small number of individuals with solid tumors in this study, assessment of death rates in relation to primary solid tumor type was not possible.

More work is needed to identify the potential impact of vaccination on COVID-19 mortality among cancer patients given recent reports suggesting differences in protection levels provided by COVID vaccines (particularly the first dose) among organ transplant and cancer patients [16,17]. Moreover, some jurisdictions in Europe and North America have elected to delay the second dose of approved COVID-19 vaccines up to 16 weeks to give more individuals a better opportunity to get their first dose. We need to confirm if this policy is safe for cancer patients given the potentially weaker immunity provided to them by the first dose of the vaccine. Likewise, some jurisdictions have elected to offer a ‘booster’ dose of vaccine to immunocompromised individuals, including some cancer patients. Whether this will decrease severe outcomes in these patients is yet to be demonstrated in a prospective study.

## Conclusion

Within the known limitations of the current study, cancer patients reported within FAERS database, who are older, males and receiving immunosuppressive treatment and those with hematological malignancies were more likely to die because of COVID-19 infection.

**Summary points**

- This study aimed to explore factors affecting coronavirus disease 2019 mortality based on a pharmacovigilance database.
- US FDA Adverse Event Reporting System quarterly data extract files were reviewed for quarters two, three and four of 2020 (i.e., April to December).
- Patients with an indication related to malignancy and a reported coronavirus disease-related reaction were selected.
- Multivariate logistic regression analysis was conducted for factors associated with a fatal outcome.
- A total of 2708 patients were included, including 721 (26.6%) patients with fatal outcomes and 1987 (73.4%) patients with nonfatal outcomes.
- The following factors were associated with fatal coronavirus disease 2019 infection: older age (odds ratio [OR]: 1.03; 95% CI: 1.01–1.04), male sex (OR: 1.43; 95% CI: 1.07–1.91), non-US source of report (OR: 2.46; 95% CI: 1.93–3.13), hematological malignancy (OR: 1.62; 95% CI: 1.28–2.07), potentially immunosuppressive treatment (OR: 1.83; 95% CI: 1.30–2.58) and diagnosis in quarter two versus quarter four (OR: 1.62; 95% CI: 1.27–2.07).

## References

[1] Kuderer NM, Choueiri TK, Shah DP Clinical impact of COVID-19 on patients with cancer (CCC19): a cohort study. Lancet 395(10241), 1907–1918 (2020). 32473681 10.1016/S0140-6736(20)31187-9 PMC7255743

[2] Woolf SH, Chapman DA, Lee JH. COVID-19 as the leading cause of death in the United States. JAMA 325(2), 123–124 (2021). 33331845 10.1001/jama.2020.24865 PMC8553021

[3] The Lancet Oncology. COVID-19 and cancer: 1 year on. Lancet Oncol. 22(4), 411 (2021). 33794199 10.1016/S1470-2045(21)00148-0 PMC8007133

[4] Zarifkar P, Kamath A, Robinson C Clinical characteristics and outcomes in patients with COVID-19 and cancer: a systematic review and meta-analysis. Clin. Oncol. 33(3), e180–e191 (2021). 10.1016/j.clon.2020.11.006 PMC7674130 33261978

[5] Carreira H, Strongman H, Peppa M Prevalence of COVID-19-related risk factors and risk of severe influenza outcomes in cancer survivors: a matched cohort study using linked English electronic health records data. EClinicalMedicine 29-30, 100656 (2020). 33437952 10.1016/j.eclinm.2020.100656 PMC7788436

[6] Choudhary S, Sreenivasulu K, Mitra P, Misra S, Sharma P. Role of genetic variants and gene expression in the susceptibility and severity of COVID-19. Ann. Lab. Med. 41(2), 129–138 (2021). 33063674 10.3343/alm.2021.41.2.129 PMC7591285

[7] Mitra P, Misra S, Sharma P. One year of COVID-19: the “new normal.” Indian J. Clin. Biochem. 36(1), 1–2 (2021). 10.1007/s12291-020-00954-x PMC7785032 33424146

[8] US FDA (2021). www.fda.gov/drugs/questions-and-answers-fdas-adverse-event-reporting-system-faers/fda-adverse-event-reporting-system-faers-public-dashboard

[9] Medical Dictionary for Regulatory Activities (2021). www.meddra.org/COVID-19-terms-and-MedDRA

[10] Liang W, Guan W, Chen R Cancer patients in SARS-CoV-2 infection: a nationwide analysis in China. Lancet Oncol. 21(3), 335–337 (2020). 32066541 10.1016/S1470-2045(20)30096-6 PMC7159000

[11] Zhang H, Wang L, Chen Y Outcomes of novel coronavirus disease 2019 (COVID-19) infection in 107 patients with cancer from Wuhan, China. Cancer 126(17), 4023–4031 (2020). 32573776 10.1002/cncr.33042 PMC7361610

[12] Meng Y, Lu W, Guo E Cancer history is an independent risk factor for mortality in hospitalized COVID-19 patients: a propensity score-matched analysis. J. Hematol. Oncol. 13(1), 75 (2020). 32522278 10.1186/s13045-020-00907-0 PMC7286218

[13] Abdel-Rahman O. Influenza and pneumonia-attributed deaths among cancer patients in the United States; a population-based study. Expert Rev. Respir. Med. 15(3), 393–401 (2021). 33107375 10.1080/17476348.2021.1842203

[14] Rogado J, Pangua C, Serrano-Montero G COVID-19 and lung cancer: a greater fatality rate? Lung Cancer 146, 19–22 (2020). 32505076 10.1016/j.lungcan.2020.05.034 PMC7260554

[15] Wu L, Zhang C, Zhao X. The impact of COVID-19 pandemic on lung cancer community. World J. Oncol. 12(1), 1–6 (2021). 33738000 10.14740/wjon1367 PMC7935618

[16] Boyarsky BJ, Werbel WA, Avery RK Immunogenicity of a single dose of SARS-CoV-2 messenger RNA vaccine in solid organ transplant recipients. JAMA 325(17), 1784–1786 (2021). 33720292 10.1001/jama.2021.4385 PMC7961463

[17] Monin-Aldama L, Laing AG, Muñoz-Ruiz M Interim results of the safety and immune-efficacy of 1 versus 2 doses of COVID-19 vaccine BNT162b2 for cancer patients in the context of the UK vaccine priority guidelines. medRxiv (2021) (Epub ahead of print).

